# Variola virus F1L is a Bcl-2-like protein that unlike its vaccinia virus counterpart inhibits apoptosis independent of Bim

**DOI:** 10.1038/cddis.2015.52

**Published:** 2015-03-12

**Authors:** B Marshall, H Puthalakath, S Caria, S Chugh, M Doerflinger, P M Colman, M Kvansakul

**Affiliations:** 1 Department of Biochemistry, La Trobe University, Kingsbury Drive, Melbourne, 3086 Victoria, Australia; 2 La Trobe Institute for Molecular Science, La Trobe University, Kingsbury Drive, Melbourne, 3086 Victoria, Australia; 3 Walter and Eliza Hall Institute of Medical Research, 1G Royal Parade, Parkville, 3052 Victoria, Australia; 4 Department of Medical Biology, University of Melbourne, Parkville, Victoria 3010, Australia

## Abstract

Subversion of host cell apoptosis is an important survival strategy for viruses to ensure their own proliferation and survival. Certain viruses express proteins homologous in sequence, structure and function to mammalian pro-survival B-cell lymphoma 2 (Bcl-2) proteins, which prevent rapid clearance of infected host cells. In vaccinia virus (VV), the virulence factor F1L was shown to be a potent inhibitor of apoptosis that functions primarily be engaging pro-apoptotic Bim. Variola virus (VAR), the causative agent of smallpox, harbors a homolog of F1L of unknown function. We show that VAR F1L is a potent inhibitor of apoptosis, and unlike all other characterized anti-apoptotic Bcl-2 family members lacks affinity for the Bim Bcl-2 homology 3 (BH3) domain. Instead, VAR F1L engages Bid BH3 as well as Bak and Bax BH3 domains. Unlike its VV homolog, variola F1L only protects against Bax-mediated apoptosis in cellular assays. Crystal structures of variola F1L bound to Bid and Bak BH3 domains reveal that variola F1L forms a domain-swapped Bcl-2 fold, which accommodates Bid and Bak BH3 in the canonical Bcl-2-binding groove, in a manner similar to VV F1L. Despite the observed conservation of structure and sequence, variola F1L inhibits apoptosis using a startlingly different mechanism compared with its VV counterpart. Our results suggest that unlike during VV infection, Bim neutralization may not be required during VAR infection. As molecular determinants for the human-specific tropism of VAR remain essentially unknown, identification of a different mechanism of action and utilization of host factors used by a VAR virulence factor compared with its VV homolog suggest that studying VAR directly may be essential to understand its unique tropism.

Variola virus (VAR), the causative agent of smallpox, is a member of the poxvirus family and belongs to the orthopoxviridae. Despite its successful eradication nearly 30 years ago, VAR remains an ongoing concern because of its potential use as a bioterrorism agent.^1^ The threat of intentional use of VAR coupled with the absence of an FDA-approved drug for the prevention or treatment of smallpox infection is cause for considerable interest in the development of small-molecule therapeutics against VAR. Current strategies for dealing with smallpox are based on vaccination using live vaccinia virus (VV),^2, 3^ a closely related member of the orthopoxvirus genus, which shares >90% sequence identity with VAR. Vaccination using live VV, however, can cause serious complications,^4^ underscoring the need for effective anti-viral treatments, particularly since anti-viral treatment may be a more efficacious strategy compared with vaccination.^5^ Recent strategies to target VAR for small-molecule therapeutics included the use of polymerase inhibitors,^6^ notably Cidofovir, inhibitors of extracellular virus formation^7^ and tyrosine kinase inhibitors including Gleevec.^8, 9^ Cidofovir is currently the only approved antiviral drug for the treatment of orthopoxviruses, although it is not approved for smallpox treatment. Other host–virus interactions have been identified that may be suitable drug targets^10, 11^ but currently require further investigation.

Several poxvirus members other than VAR have been shown to rely on virulence factors that prevent premature host cell demise via programmed cell death or apoptosis,^12, 13, 14, 15, 16^ thus ensuring survival and proliferation. The B-cell lymphoma 2 (Bcl-2) protein family is a key mediator for maintaining cell survival or to drive apoptosis, thereby removing infected, damaged or unwanted cells,^17^ and sequence, structural and functional orthologs of Bcl-2 have been found in a number of poxviruses.^18^ Certain viral Bcl-2-like proteins were only identified as family members after their 3D structures were determined, owing to their complete lack of sequence identity to mammalian Bcl-2 proteins. This group of proteins include the myxoma virus M11L^12^ and VV F1L^15^ and N1L.^19^ Myxoma virus M11L was shown to adopt the classical Bcl-2 fold^20, 21^ that utilizes the canonical Bcl-2 homology 3 (BH3)-binding groove to engage BH3 ligands to exert its pro-survival effect. VV F1L also adopts a Bcl-2 fold, but unlike M11L it exists as a domain-swapped dimer,^22, 23^ whereas N1L also adopted a dimeric Bcl-2 fold but with a different dimeric arrangement.^24, 25^

Although F1L from VAR has not previously been investigated, the VV homolog is well characterized. VV F1L has been shown to inhibit the mitochondrial pathway of apoptosis by replacing Mcl-1^26^ and interacts with the isolated BH3 domains of Bim, Bax and Bak,^23^ which are bound in the canonical Bcl-2-binding groove.^22^ Furthermore, an F1L-deficient VV potently causes Bak/Bax-mediated apoptosis.^15, 27^ Functionally, VV F1L appears to rely primarily on neutralization of Bim in the context of a viral infection.^22^ Given the close similarity between VAR and VV, VAR may also rely on inhibition of host cell apoptosis for successful infection and proliferation. Disruption of VAR ability to inhibit apoptosis thus may constitute an attractive strategy for small-molecule-based intervention. To investigate this possibility, we performed a biochemical, structural and functional characterization of VAR F1L. Here we report that despite possessing a nearly identical 3D structure and sequence, VAR F1L inhibits apoptosis via a different mechanism compared with its homolog in VV.

## Results

**Figure 1:**
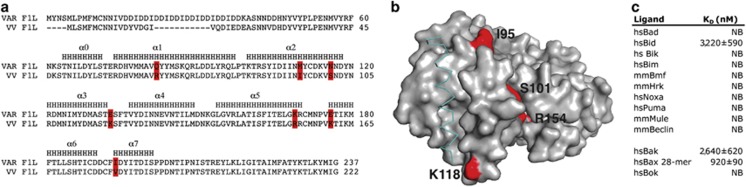
(**a**) Sequence alignment of vaccinia virus (VV) with variola virus (VAR) F1L. Red denotes sequence differences in the Bcl-2 domain, H indicates helices. (**b**) Sequence variations between VV and VAR F1L are mapped in red on a molecular surface of VV F1L (gray). (**c**) BH3-binding profile of VAR_F1L. *K*_D_ values were determined by isothermal calorimetry

Given the high sequence identity of VAR F1L with VV F1L (Figure 1a) and the location of primary sequence variations (all of which are away from the identified BH3-domain-binding groove in VV F1L (Figure 1b and Supplementary Figure 1)), we surmised that VAR F1L would display a ligand-binding behavior similar to VV F1L. Using recombinant VAR F1L we tested a panel of pro-apoptotic BH3-domain ligands using isothermal calorimetry (Figure 1c). Previously, we showed that VV F1L exhibited a highly selective BH3-domain ligand-binding profile, with high affinity for pro-apoptotic Bim (*K*_D_=200 nM), and considerably lower affinity for Bax and Bak BH3 domains (*K*_D_ of 2000 and 4300 nM, respectively).^23^ Similarly, VAR F1L bound Bax and Bak BH3 domains weakly with *K*_D_ values of 920 and 2640 nM, respectively. Unexpectedly, VAR F1L showed no detectable affinity for Bim BH3, and only weakly bound Bid from the BH3-only proteins with a *K*_D_ of 3220 nM. Considering the weak affinity of VAR F1L for Bax and Bak BH3 ligands, we then investigated if VAR F1L is able to engage and restrain full-length Bax and Bak. For this purpose, we utilized a yeast-based assay in which we expressed full-length Bak, Bax and VAR F1L. The assay is based on the observation that Bax or Bak overexpression is lethal in yeast, but can be repressed by co-expression of Bcl-2, Bcl-x_L_, Mcl-1 or A1. Similar to endogenous mammalian pro-survival Bcl-2, VAR F1L is able to prevent both Bak- and Bax-induced yeast death (Supplementary Figure 2), indicating that VAR F1L is able to directly engage both Bak and Bax. In contrast, VV F1L has only been shown to bind Bak but not Bax in yeast (Supplementary Figure 2), as previously observed in mammalian cells.^28^

### Structural basis for VAR F1L BH3 domain ligand binding

**Figure 2:**
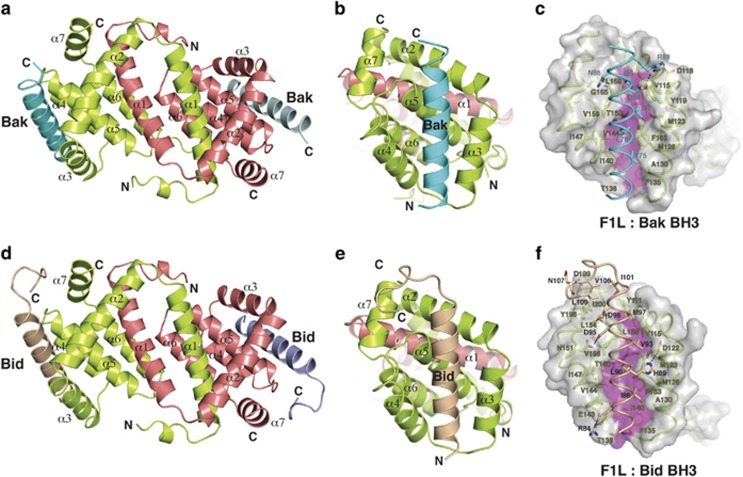
Structures of VAR F1L:Bak and Bid BH3 complexes. (**a**) Cartoon diagram of F1L:Bak BH3 complex. F1L chains are shown as a cartoon (lime and salmon), with helices *α*1-7 labeled. Two Bak BH3 chains are show in cyan and light blue. (**b**) Cartoon diagram of F1L:Bak BH3 complex. This view looks into the hydrophobic BH3-binding grove, which is formed by helices α2–5. F1L helices α2–7 from monomer 1 (lime) are labeled, as is helix α1′ from monomer 2 (salmon). Bak BH3 is shown in cyan. (**c**) Cartoon diagram of F1L:Bid BH3 complex. F1L (lime and salmon) in complex with Bid BH3 (wheat and lilac). The view is as in **a**. (**d**) Cartoon diagram of F1L:Bid BH3 complex. This view is as in **b**. F1L helices α2–7 from monomer 1 (lime) are labeled, as is helix α1′ from monomer 2 (salmon). Bid BH3 is shown in wheat. (**e**) Stereo diagram of the F1L (lime):Bak (cyan) complex interface. The F1L surface is shown in gray, except for magenta shading indicating the floor of the peptide-binding groove. F1L residues are labeled in lime, Bim BH3 residues are labeled in cyan. (**f**) Stereo diagram of the F1L (lime):Bim (wheat) complex interface. View and labeling is as in **c**, except for labeling of Bid residues (in black)

To investigate the molecular basis for Bid, Bak and Bax BH3 domain binding by VAR F1L, we determined crystal structures of VAR F1L in complex with Bak and Bid BH3 domain peptides (Figures 2a and d, Table 1). As observed previously for VV F1L,^22, 23^ VAR F1L adopts a Bcl-2-like fold that forms a domain-swapped Bcl-2 with similar binding grooves that engage BH3-domain ligands (Figures 2b and e). Superimposition of bound Bak and Bid BH3 backbones indicate that both ligands are bound in near identical manner (Supplementary Figures 3a and b). In the VAR F1L:Bak BH3 complex (Figure 2c), residues V75, L78, I81, I85 protrude into four pockets in the VAR F1L-binding groove, similar to the equivalent hydrophobic residues in Bid (Figure 2f; I86, L90, V93, M97).

### Comparison of VAR_F1L:Bak BH3 with VV_F1L:Bak BH3 complex

Superimposition of VV F1L with VAR F1L from their respective complexes with Bak BH3 yields a root-mean-square deviation of 0.9 Å over 138 C*α* atoms, with the only notable differences in side chain orientations between VV F1L and VAR F1L occurring at F135 (Supplementary Figure 4). Overall, the VV F1L and VAR_F1L complexes with Bak appear to be near identical.

### VAR F1L only inhibits Bax but not Bak-mediated apoptosis

**Figure 3:**
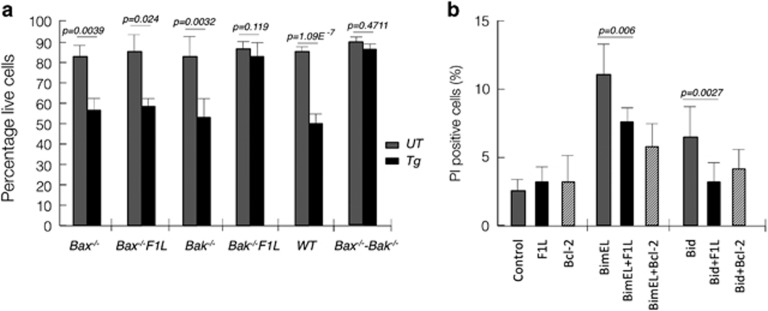
VAR F1L inhibits Bax- but not Bak-mediated apoptosis. (**a**) Viability of wild-type, Bax^−/−^, Bak^−/−^ and Bax^−/−^/Bak^−/−^ DKO cells. MEF cells stably overexpressing VAR F1L or vector, treated with 1.5 *μ*M thapsigargin and cultured for 24 h. (**b**) Viability of 293T cells transiently overexpressing either Bim_EL_ or Bid as well as F1L, Bcl-2 or vector control. Error bars are ±S.E.M. with *n*=3

To identify differences in anti-apoptotic activity between VAR and VV F1L, we retrovirally transfected VAR F1L into wild-type, Bax^−/−^, Bak^−/−^ and Bax^−/−^/Bak^−/−^ DKO cells and treated them with thapsigargin to induced apoptosis via ER stress. Unexpectedly, VAR F1L was unable to protect both wild-type and Bax^−/−^ mouse embryonic fibroblasts (MEFs), whereas Bak^−/−^ MEFs were efficiently protected from apoptosis (Figure 3a) in a manner similar to Bcl-2 (Figure 3b), suggesting that VAR F1L exclusively inhibits Bax-mediated apoptosis. Similar results were obtained after serum withdrawal, with VAR F1L maintaining viability of Bak^−/−^ MEFs but not wild-type and Bax^−/−^ MEFs (Supplementary Figure 5). Overall, the cellular assays mirror our peptide-binding data obtained by ITC, where the lower affinity ligand Bak is not inhibited in a cellular context. Considering the peptide-binding data, we next investigated whether VAR F1L is able to protect against Bim- and Bid-induced apoptosis. 293T cells were transfected with either Bim_EL_ or Bid. VAR F1L protected against both Bim_EL_ (*P*=0.006) or Bid-mediated apoptosis (*P*= 0.0027), with comparable levels of protection observed with Bcl-2.

## Discussion

Interactions between pro- and anti-apoptotic Bcl-2 family members are critical for the regulation of apoptosis during normal development as well as during disease states including viral infections or cancer.^17^ Compelling evidence points to a critical role for viral Bcl-2 proteins during the viral life cycle, particularly viral infectivity and proliferation.^12, 15, 29^ No anti-apoptotic activity for VAR encoded proteins has been demonstrated to date, although a number of proteins from the closely related VV have been reported to manipulate host cell death signaling.^15, 30^ We have now shown biochemically that VAR F1L is able to engage a limited number of pro-apoptotic Bcl-2 proteins including Bid, Bak and Bax. This binding profile is highly unusual, as all reported cellular or viral anti-apoptotic Bcl-2 family members engage Bim,^31^ the sole pan pro-survival antagonist of the Bcl-2 family,^32^ making VAR F1L the first reported exception. This observation was unexpected as VV F1L binds Bim BH3 *in vitro* and *in vivo.*^22, 28^ Furthermore, VV F1L prevents both Bak- and Bax-mediated apoptosis in cell-based assays,^26, 28^ whereas VAR F1L was able to neutralize only Bax-mediated apoptosis. Close examination of structures of VAR F1L complexes with Bid and Bak and VV F1L complexes with Bim and Bak did not reveal major differences in BH3 domain binding to the canonical binding groove (Supplementary Figure 4) that can explain the startlingly different ligand-binding behavior. The loss of Bim binding for VAR F1L is particularly noteworthy as VV-induced apoptosis is reduced in Bim-deficient cells,^28^ suggesting that Bim plays a substantial role during the cellular response to VV infection. Whether or not Bim is important for VAR infection has not been determined to date. In the case of Epstein–Barr virus BHRF1-mediated inhibition of apoptosis, Bim^33^ and Bak^34^ neutralization were shown to be critical, whereas in the case of myxoma virus M11L,^20^ Bax and Bak sequestration were identified as the primary mechanism of inhibition. However, in both cases, the mechanism of apoptosis inhibition was not defined in the context of a viral infection. Nonetheless, despite the inability to directly engage Bim, and the very modest affinity to Bid, VAR F1L is able to counter both Bim- and Bid-mediated apoptosis.

In addition to raising intriguing questions about VAR biology, this striking insensitivity of VAR F1L to Bim raises questions about the regulation of Bcl-2-mediated apoptosis. Direct activation of Bax and Bak via promiscuous or activator BH3-only proteins has been shown to be an important route to trigger apoptosis,^35, 36, 37, 38^ however, VAR F1L appears to be unlikely to act by BH3-only protein sequestration to prevent Bax activation. Among the other alternatively proposed mechanisms invoked to control apoptosis, VAR F1L's ability to inhibit apoptosis is most readily explained by an indirect model based on sequestration of Bax and Bak,^39, 40^ where Bax and Bak are held in an inactive configuration by a pro-survival Bcl-2 family member. This is supported by our observations that VAR F1L is able to bind BH3 domain peptides from Bax and Bak, albeit at modest affinity, as well as the prevention of Bax- and Bak-induced growth arrest in yeast-based assays in the absence of any other cellular apoptosis regulatory proteins. Such a mechanism is also an integral part of the currently favored unified model,^41^ where a balance between direct activation and indirect activation ultimately yields control of intrinsic apoptosis. The insensitivity of VAR F1L to Bim, and indeed all tested BH3-only proteins, is striking in this context, and may make VAR F1L a useful tool molecule to further understand the interplay of the two main modes that underpin the unified model of apoptosis regulation.

Targeting of the apoptotic machinery is currently an intensely pursued strategy for the development of novel drugs for cancer therapy.^42, 43^ Similarly, VAR F1L may be a valid target for development of antiviral therapeutics. Current strategies for the development of antiviral therapeutics against VAR have relied mostly on the use of homologous target molecules from VV, monkeypox or ectromelia virus, with only a small number of studies utilizing VAR-encoded proteins,^44, 45, 46^ and even fewer studies using their structures.^47, 48^ The marked differences between VAR and VV F1L suggest that studies of VAR proteins may provide unexpected avenues for therapeutic intervention that may not be predicted based on available data from the closely related and much better understood VV. It also raises the question of how well current systems that mimic VAR model the behavior of this intriguing virus. VAR occupies a unique position as the sole human pathogen among the orthopoxviruses. As molecular determinants for this human-specific tropism remain essentially unknown, identification of a different mechanism of action and utilization of host factors used by a VAR virulence factor compared with its VV homolog suggest that studying VAR directly may be essential to understand its unique tropism.

## Materials and Methods

### All experiments were performed with WHO approval

#### Recombinant proteins and binding experiments

VAR_F1LΔN38ΔC36 was amplified by PCR from expression-optimized F1L cDNA (Blue Heron), cloned into the pGEX-6P3 vector (Invitrogen, Melbourne, VIC, Australia) using *Bam*HI and *Eco*RI, and expressed in *E. coli* BL21 DE3 pLysS cells. Cells were homogenized using an Avestin EmulsiFlex homogenizer in lysis buffer (50 mM Tris-HCl, pH 8.0, 150 mM NaCl, 1 mM EDTA). After centrifugation, the supernatant was applied to a glutathione sepharose column (GE Healthcare, Melbourne, VIC, Australia) and washed with lysis buffer. On-column cleavage was performed using Prescission Protease (GE Healthcare), and VAR F1L was eluted using lysis buffer. Subsequently, VAR F1L was subjected to gel-filtration chromatography in 25 mM HEPES, pH 7.5, 150 mM NaCl using a Superdex 200 column (GE Healthcare). Calorimetry data were collected on a VP-ITC (MicroCal) with VAR_F1LΔN38ΔC36 as previously described.^23^ Peptides used have been described previously.^34^

#### Crystallization and structure determination

VAR F1L:Bak or VAR F1L:Bid BH3 complexes were obtained by mixing VAR F1L with human Bak 26-mer or Bid 34-mer peptide in a 1:1.25 molar ratio and concentrated using a centricon (Millipore) to 5 mg/ml.

VAR F1L:Bak BH3 crystals were grown by the sitting drop method at room temperature in 1.7 M MgSO_4_, 0.1 M sodium acetate with pH 5.2.The crystals belong to space group I121 with *a*=124.774 Å, *b*=68.762 Å, *c*=171.598 Å, α=90°, *β*=109.43°, *γ*=90°. The asymmetric unit contains six VAR F1L chains and six Bak BH3 peptides, with 49% solvent content. Diffraction data were collected from crystals flash frozen in paratone at 100 K using beamline MX2 at the Australian Synchrotron (Melbourne, VIC, Australia).

VAR F1L:Bid BH3 crystals were grown by the sitting drop method at room temperature in 1.8 M sodium acetate and 0.1 M HEPES (pH 6.5). The crystals belong to space group I121 with *a*=124.774 Å, *b*=68.762 Å, *c*=171.598 Å, *α*=90°, *β*=109.43°, *γ*=90°. The asymmetric unit contains one VAR F1L chain and one Bid BH3 peptides, with 49% solvent content. Diffraction data were collected from crystals flash frozen in paratone at 100 K using beamline MX2 at the Australian Synchrotron.

Diffraction data were processed with XDS^49^ and programs of the CCP4 suite.^50^ The VAR F1L:Bak BH3 and VAR F1L:Bid BH3 structures were solved by molecular replacement with PHASER^51^ using F1L from the VV F1L structure as a search model (PDB 2VTY) and refined using Phenix.^52^ All data collection and refinement statistics are summarized in Supplementary Table 1. Figures were prepared using PyMol.^53^ Software was in some instances accessed via SBGrid.^54^

#### Yeast colony assays

*Saccharomyces cerevisiae* W303*α* cells were co-transformed with pGALL(TRP) vector only, pGALL(TRP)-Bcl-x_L_, or pGALL(TRP)-VAR_F1L and pGALL(Leu)-Bak or pGALL(Leu)-Bax. pGALL(TRP) and pGALL(Leu) places genes under the control of a galactose inducible promoter. Cells were spotted as fivefold serial dilutions onto medium containing 2% (w/v) galactose (inducing, ‘ON'), which induces protein expression, or 2% (w/v) glucose (repressing, ‘OFF'), which prevents protein expression, as previously described.^55^ Plates were incubated for 48 h at 30 °C and then photographed.

#### Cell culture, transfection, lentiviral infection and cellular assays

Human embryonic kidney 293T (ATCC CRL-3216) and MEFs (a gift from D Huang) were cultured in Dubecco's modified Eagle's medium supplemented with 10% fetal calf serum (Sigma) at 37 °C in a humidified 10% CO_2_ incubator. Both *bak*^−/−^ and *bax*^−/−^ MEFs were generated from E15 embryos in accordance with standard procedures and were infected with SV40 large T antigen-expressing lentiviruses as described.^39, 40^

To generate lentiviral particles, human embryonic kidney 293T cells were transfected with packaging constructs pCMV ðR8.2 and VSVg and the relevant lentiviral plasmid at a ratio of 1:0.4:0.6 using Fugene 6.0 transfection reagent (Roche Lifesciences, Indianapolis, IN, USA) following the manufacturer's instructions. The virus containing supernatants were harvested, filtered (0.8 *μ*m) and supplemented with polybrene (4 mg/ml). Target cells were infected with virus supernatant as described by Vince *et al.*^56^

MEF cells were seeded at ~10 000 cells per well and allowed to adhere for 24 h. The cells were then treated with Thapsigargin (1.5 *μ*M final, Calbiochem, San Diego, CA, USA) or serum withdrawal for 24 and 48 h. To monitor cell death, cells were stained with propidium iodide (1.25 *μ*g/ml) and analyzed in a FACScan (Becton Dickinson). Values presented are the means±S.D. (*n*=3). Differences were considered significant at *P*<0.05.

293T cells were seeded @150 000 cells per well in six-well plates in complete Dubecco's modified Eagle's medium and incubated O/N @37 °C, 5% CO_2_. Next day, the cells were transfected with 0.25 *μ*g Bim, Bid or Bcl2 and 0.5 *μ*g F1L or control plasmid DNA per well using Xtreme Gene 9 transfection reagent (Roche Lifesciences; Catalog number 06365787001). 24 h post transfection, cells were harvested by trypsinization and PI uptake measured by flow cytometry. Error bars presented are the standard error of the means (*n*=3).

## Figures and Tables

**Table 1:** Crystallographic statistics

*Crystal*	*F1L: Bak*	*F1L: Bid*
*Data collection and phasing*		
Spacegroup	I2	F222
Resolution range (Å)	40 - 2.55	50 - 1.75
Unique reflections	45043	17113
Multiplicity^a^	4.1 (4.1)	13.3 (9.2)
Completeness (%)^a^	99.9 (100.0)	97.1 (80.4)
*R*_merge_^a^^,^^b^	0.075 (0.656)	0.062 (0.459)
Mn *I*/*σI*	12.0 (2.2)	39.0 (4.1)
		
*Refinement*		
Resolution range (Å)	39.7 - 2.55	40.5 - 1.75
Reflections (working set/test set)	41807/2127	15817/842
Protein atoms	7943	1351
Solvent atoms	98 H_2_O	91 H_2_O
*R*_cryst_/*R*_free_^c^	0.197/0.242	0.172/0.213
r.m.s.d. bonds (Å)	0.002	0.012
r.m.s.d. angles (º)	0.5	1.2
Ramachandran plot (%)^d^	95.1/4.9/0.0/0.0	98.0/2.0/0.0/0.0

a Numbers in parentheses refer to the highest resolution shells.

b *R*_merge_=Σ_*h*_Σ_*i*_ | *I*_*i*_(*h*) - <*I*(*h*)> |/Σ_*h*_Σ_*i*_*I*_*i*_(*h*), where *I*_*i*_(*h*) is the *i*th measurement of reflection *h* and <*I*(*h*)> is the weighted mean of all measurements of *h*.

c R=Σ_*h*_|*F*_obs_ - *F*_calc_|/Σ_*h*_*F*_obs_, where *F*_obs_ and *F*_calc_ are the observed and calculated structure factor amplitudes, respectively. *R*_cryst_ and *R*_free_ were calculated using the working and test set, respectively.

d Residues in most favoured, additionally allowed, generously allowed and disallowed regions

## Abbreviations

Bcl-2: B-cell lymphoma 2
VAR: variola virus
VV: vaccinia virus
BH: Bcl-2 homology
MEF: mouse embryonic fibroblast
