# Supplementary material for: Variola virus F1L is a Bcl-2-like protein that unlike its vaccinia virus counterpart inhibits apoptosis independent of Bim
Source: Cell Death Dis. 2015 Mar 12;6(3):e1680–. doi: 10.1038/cddis.2015.52 (PMC4385930; doi:10.1038/cddis.2015.52)
Supplement: Supplementary Information [file cddis201552x1.docx]

**Supplementary information**

**Figure S1:** **Sequence variations between VAR and VV F1L.** Molecular surface of VV F1L is shown in grey, with sequence variations to VAR F1L shown in red. **a)** Left-hand view into the canonical binding groove. **b)** Right-hand view into the canonical binding groove. **c)** Bottom view along the canonical binding groove.

**Figure S2:** **VAR F1L is able to directly engage Bax and Bak.** Yeast co-transformed with constructs encoding Bax or Bak and the indicated pro-survival proteins, each under the control of an inducible (GAL) promoter, were spotted onto inducing galactose (‘‘ON’’) plates as 5-fold serial dilutions. Image is representative of 2 independent experiments.

**Figure S3:** **Comparison of VAR F1L:Bak and Bid BH3 complexes.**

**a)** BH3 domain binding to VAR F1L. Bak (cyan) and Bid (dark blue) BH3 domains are shown as traces bound to the hydrophobic binding groove on F1L shown as a molecular surface (grey). **b)** Superimposition of the VAR F1L main chains from the two complexes formed with Bak (lime) and Bid (dark blue) BH3 domains. **c)** Superimposition of VV (magenta) and VAR F1L (lime) residues that form the canonical hydrophobic binding groove. Key F1L residues are labeled in black. Bak BH3 from VAR F1L (cyan) and VV F1L (orange) are shown as cartoon, with key binding residues shown as sticks.

**Figure S4:** **Detailed view of the VAR F1L:Bid and VV F1L:Bim complexes. a)** The VAR F1L binding groove as seen from helix α3. VAR F1L surface is shown in grey, with the bottom of the canonical binding groove shown in magenta. Bid BH3 is shown as a ribbon (wheat). 4 key hydrophobic residues are shown bound to their respective binding pockets. **b)** The view is as shown in A. VV F1L surface is shown in grey, with the bottom of the canonical binding groove shown in orange. Bim BH3 is shown as a ribbon (salmon).

**Figure S5: Variola virus F1L protects against apoptosis induced by serum withdrawal.** Viability of wild-type, Bax^-/-^, Bak^-/-^ and Bax^-/-^/Bak^-/-^ DKO cells MEF cells stably overexpressing F1L or vector, after serum withdrawal up to 72 hrs.
