# Supplementary figures and images for: Variola virus F1L is a Bcl-2-like protein that unlike its vaccinia virus counterpart inhibits apoptosis independent of Bim
Source: Cell Death Dis. 2015 Mar 12;6(3):e1680–. doi: 10.1038/cddis.2015.52 (PMC4385930; doi:10.1038/cddis.2015.52)

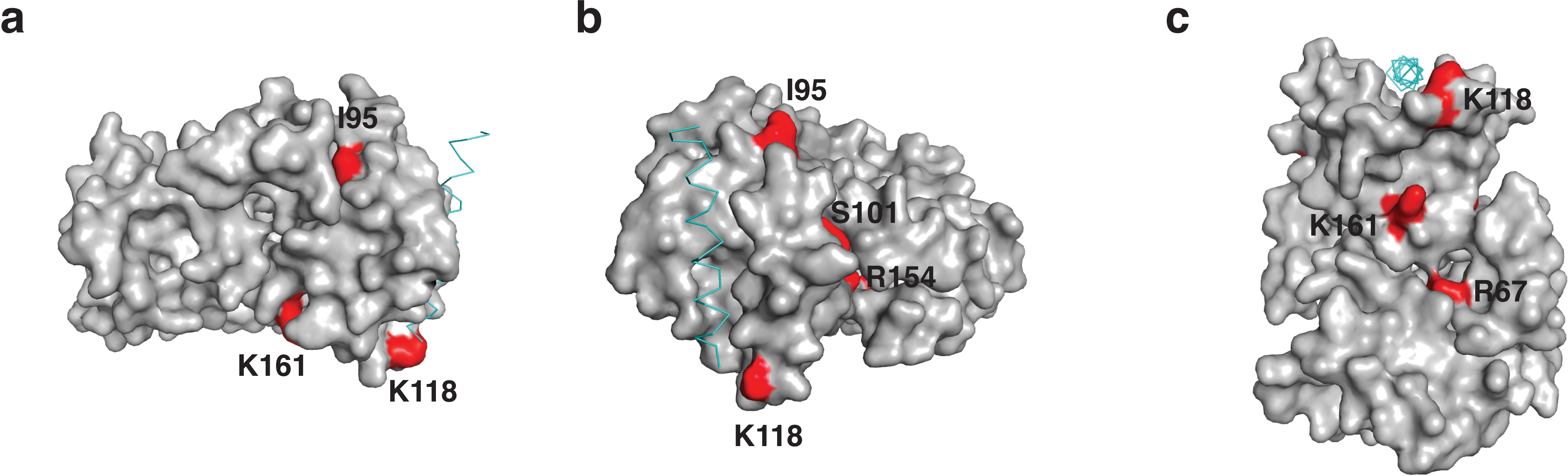

Supplement: Supplementary Figure 1 [file cddis201552x2.tif]

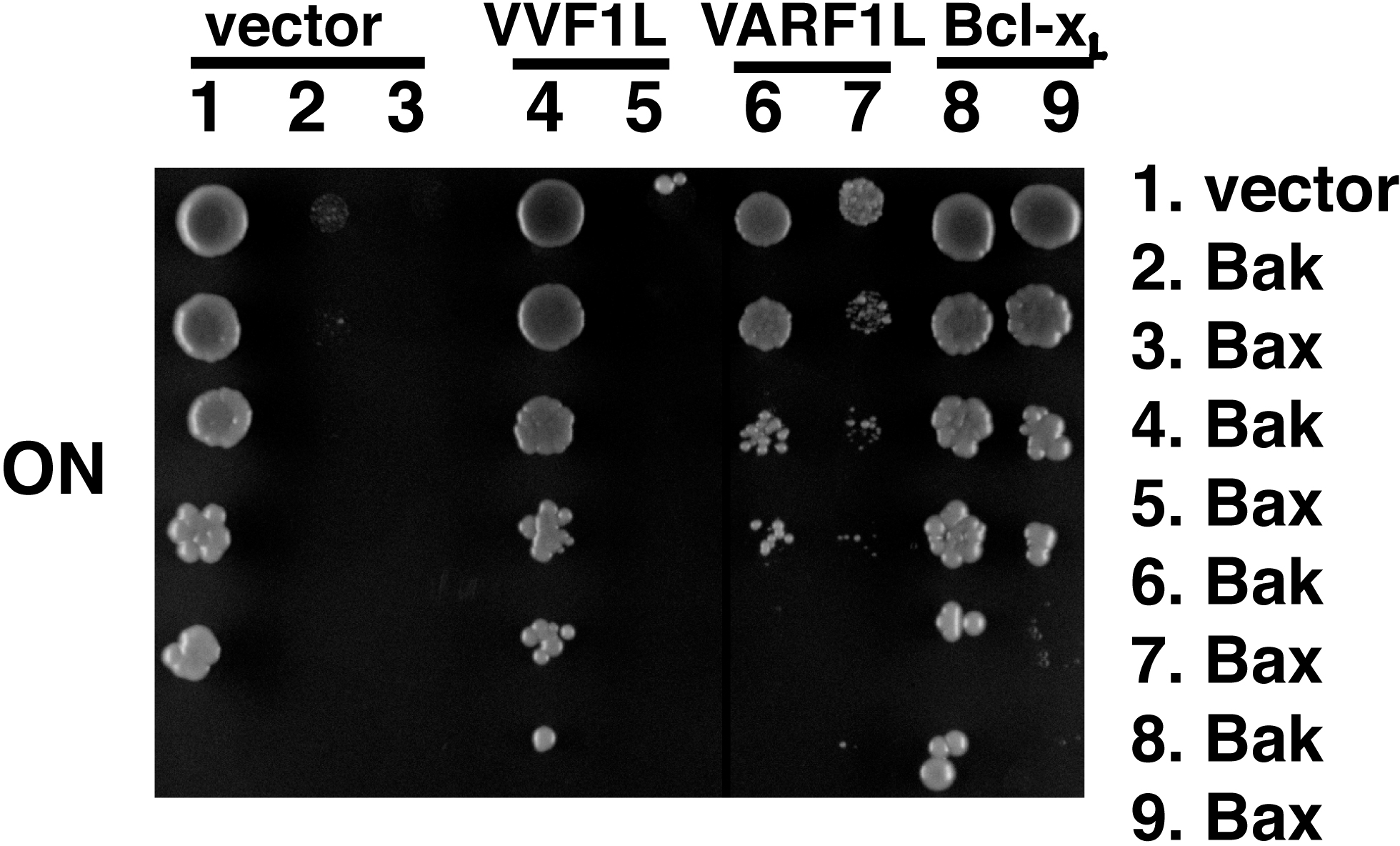

Supplement: Supplementary Figure 2 [file cddis201552x3.tif]

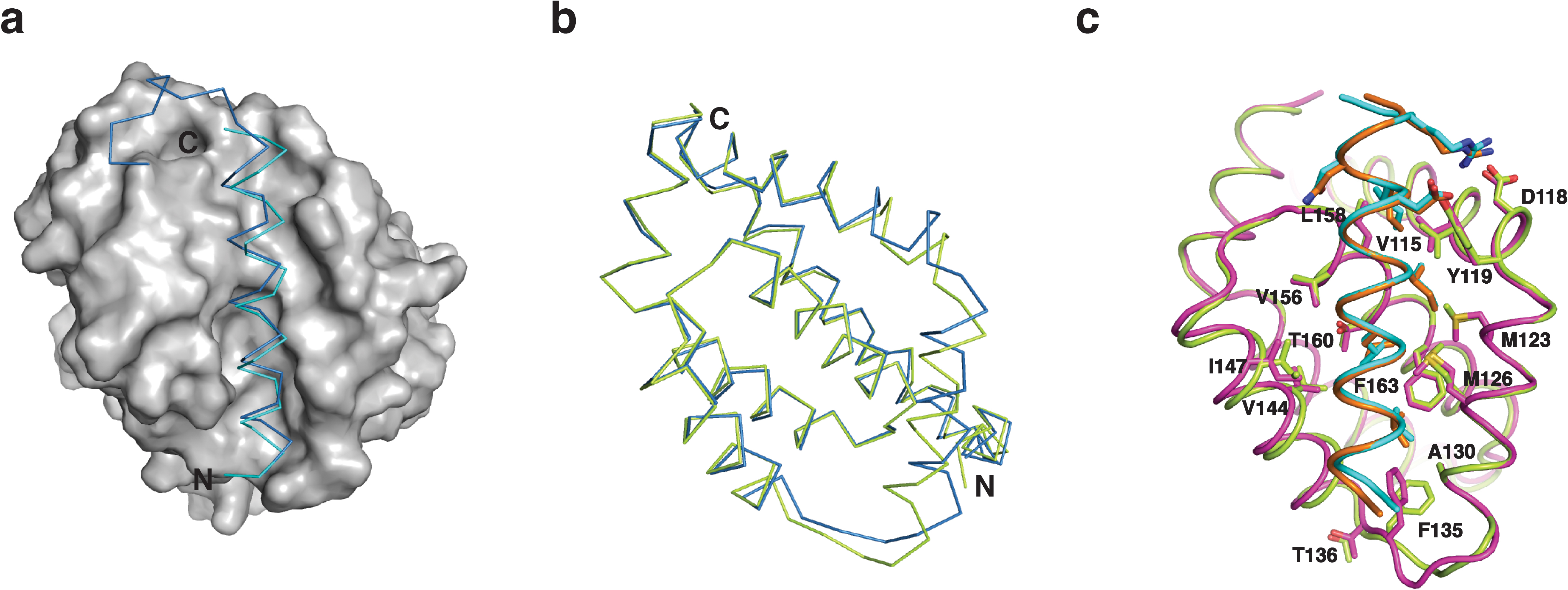

Supplement: Supplementary Figure 3 [file cddis201552x4.tif]

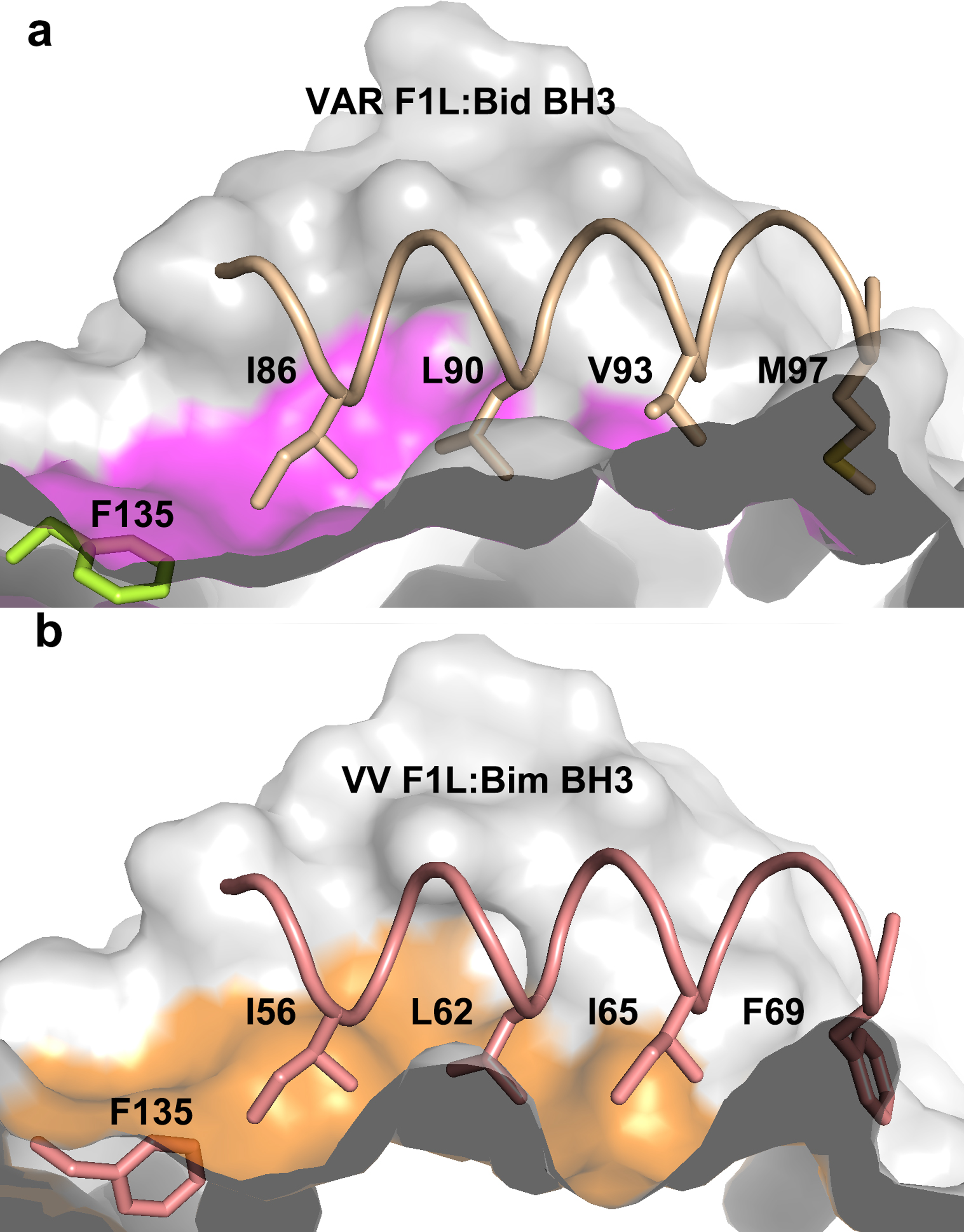

Supplement: Supplementary Figure 4 [file cddis201552x5.tif]

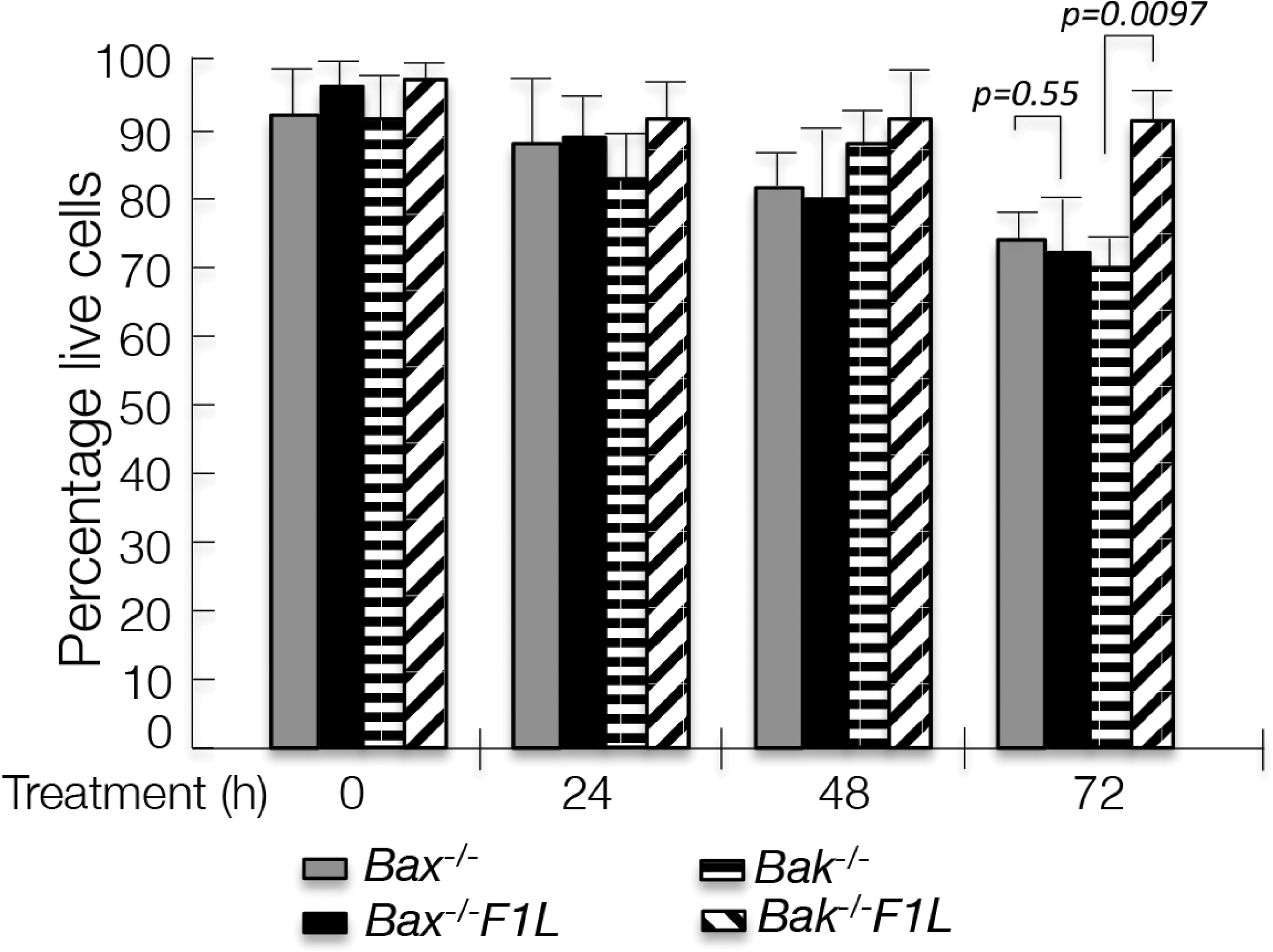

Supplement: Supplementary Figure 5 [file cddis201552x6.tif]
